# The impact of antibiotic prophylaxis with intracameral cefuroxime on postoperative infectious endophthalmitis rates in a high-volume cataract surgery center

**DOI:** 10.1038/s41598-023-45398-4

**Published:** 2023-10-21

**Authors:** Luciane Nunes de Sousa Casavechia, Antonio Carlos Meireles, Evandro Schapira, Rodrigo Antonio Brant Fernandes, Arthur Gustavo Fernandes

**Affiliations:** 1 Ophthal Hospital Especializado, Avenida Ministro Gabriel de Resende Passos, 500, Sao Paulo, SP CEP: 04521-020 Brazil; 2 Department of Ophthalmology and Visual Sciences, Paulista Medical School, Federal University of São Paulo – UNIFESP, São Paulo, SP Brazil; 3 Roski Eye Institute, Keck School of Medicine, University of Southern California, Los Angeles, CA USA; 4 Department of Anthropology and Archaeology, University of Calgary, Calgary, AB Canada

**Keywords:** Infection, Health care

## Abstract

Our purpose was to compare postoperative infectious endophthalmitis rates before and after the introduction of antibiotic prophylaxis via intracameral with cefuroxime (ATB-P IC) in a high-volume cataract surgery service. Retrospective cohort study considering patients who underwent cataract surgery at Ophthal Hospital Especializado, São Paulo, Brazil, from January/2011 to December/2019. Patients operated from 2013 to 2019 comprised the ATB-P IC group while those operated from 2011 to 2013 formed the control group without the ATB-P IC protocol. A total of 23,184 cataract surgeries were included, with 6,207 in the Control Group and 16,977 in the ATB-P Group. A significantly higher rate of endophthalmitis was observed in the control group (0.0967%) when compared to the ATB-P group (0.0177%) (*p* = 0.014). Surgeries performed with ATB-P showed 80% less chance of reported endophthalmitis (OR = 0.20; 95% CI 0.05–0.72; *p* = 0.014) than those without ATB-P. Of the six cases confirmed by culture in the control group, all tested positive for *Pseudomonas aeroginosa* and the only case confirmed by culture in the ATB-P group was positive for *Staphylococcus epidermidis.* Our findings strongly support the use of intracameral antibiotic prophylaxis with cefuroxime to reduce postoperative infectious endophthalmitis rates, and we recommend its incorporation into cataract surgery protocols.

## Introduction

Cataract surgery is a procedure with low rates of complication, however, complications associated with intraocular infection can significantly compromise post-operative visual outcomes. The eye inflammation caused by external pathogens during cataract surgery is classified as infectious endophthalmitis. Acute-onset postoperative endophthalmitis is defined as an infection that manifests within 6 weeks after an eye procedure. However, most patients with postoperative endophthalmitis present an early onset clinical picture, typically in the immediate postoperative period up to seven days after surgery^1^. The clinical presentation progresses with symptoms such as visual loss, pain, redness, and intraocular inflammation^2^. The inflammatory reaction of tissues and/or ocular fluids caused by acute infectious endophthalmitis is rare, with an estimated incidence ranging from 0.012 to 1.3%^3,4^. Nevertheless, it is one of the most serious and feared complications because, in addition to prolonging treatment time and costs, it can lead to irreversible morbidities^5^.

The main cause of postoperative infectious endophthalmitis is associated with the local microbiota, which enters the eye mainly during or shortly after the surgery, especially through insufficiently sealed incisions^2,3,6^. In this regard, it is recommended to address any local abnormalities such as blepharitis, conjunctivitis, canaliculitis, dacryocystitis, lacrimal duct obstruction, or systemic conditions like immunosuppression, diabetes mellitus, distant infections, before the surgical procedure^3,7,8^. Additionally, measures to ensure the sterility of materials, the selection of the appropriate surgical technique, and adequate asepsis and antisepsis, such as eyelashes isolation, the use of steridrapes, and the instillation of povidone-iodine eye drops into the conjunctival sac, are essential^4,9,10^.

Cataract surgery is classified as clean procedure, and therefore, antimicrobial prophylaxis would not be typically used as the risk of infection is low. Clean surgeries are recommended to adopt antimicrobial prophylaxis only in cases of prosthesis placement^6^. Antibiotic prophylaxis (ATB-P) assumes the presence of sufficient serum and tissue levels at the time of incision, which will be maintained until the end of the operative procedure^6^. Taking it into account, there would be greater bioavailability and antibiotic concentration via topical administration in the immediate preoperative phase and via intracameral (IC) during the intraoperative phase. Various guidelines for preventing infectious endophthalmitis support the use of topical povidone-iodine before the surgical procedure in conjunction with IC injection of cefuroxime formulations (Aprokam® – Laboratoires Théa, Clermont-Ferrand, France) at the end of the procedure^11,12^.

The choice of antibiotic should be guided by the most prevalent microorganisms causing endophthalmitis. The most common bacteria are Gram-positive species, such as coagulase-negative *Staphylococcus*^1,7,8,11,13^; while Gram-negative bacteria like *Pseudomonas* and fungal infections are less common^3,11,13^. In that sense, the most commonly used antibiotics for intraocular prophylaxis are cefuroxime, moxifloxacin and vancomycin^11^. Cefuroxime, a second-generation cephalosporin, was initially studied for IC prophylaxis by Montan et al. in the early 1990s^11^ and offers broad coverage of Gram-positive and some Gram-negative aerobic organisms^11,13–15^. A 2006 prospective, randomized, controlled trial conducted by the European Society of Cataracts and Refractive Surgeons reported a 20% reduction in endophthalmitis rates compared to surgeries without such prophylaxis^13^.

While numerous studies have assessed the safety and efficacy of intracameral antibiotic prophylaxis, there is a limited body of research conducted in Brazil, with a predominant focus on moxifloxacin^16^. It's worth noting that the causative agents of endophthalmitis can vary based on population demographics, geographic factors, and lifestyle^17^, underscoring the importance of studies that encompass diverse groups of individuals. The purpose of the current study was to compare the endophthalmitis rates before and after the introduction of the ATB-P protocol with intracameral cefuroxime in a high-volume cataract surgery service in Brazil.

## Methods

This retrospective cohort study included all patients who underwent cataract surgery at the Ophthal Cataract Outpatient Clinic—Hospital Especializado, São Paulo, Brazil, between January 2011 and December 2019. Data were obtained from the electronic medical records of all operated patients. The study protocol was approved by the H Olhos Paulista Research Ethics Committee and written informed consent was waived by the same committee. All methods were carried out in accordance with the tenets of the Declaration of Helsinki.

Patients were categorized into 2 groups based on the year of surgery: patients operated from 2013 to 2019 constituted the ATB-P group, in which the intracameral antibiotic prophylaxis protocol was applied during their surgeries; patients operated from 2011 to 2013 comprised the control group, where no intracameral antibiotic prophylaxis protocol was applied. Exclusion criteria included loss of postoperative follow-up, combined surgeries, secondary intraocular lens implantation, patients unable to receive intraoperative ATB-P (e.g. due to allergy), cases of endophthalmitis arising from other surgeries, endophthalmitis in patients referred from other services, and cases of endogenous and chronic endophthalmitis.

On the day of surgery, all patients went through the following protocol: upon arriving in the operating room, patients had the eyelid region cleansed with a germicidal agent, Chlorhexidine, and received eye drops of anesthetic, mydriatic and 5% iodine 30 min before the surgery, except for patients allergic to iodine, who received only eyelid hygiene with Chlorhexidine along with the anesthetic and mydriatic eye drops. After sedation, 5% povidone-iodine eye drops were instilled approximately 5 min before the surgical procedure commenced. The standard asepsis and antisepsis procedures with povidone-iodine or chlorhexidine were carried out. At the end of the cataract surgery, patients in the ATB-P group received a 1 cc intracameral cefuroxime solution with a concentration of 1 mg/0.1ml and 2 drops of topical moxifloxacin eye drops. The cefuroxime solution was prepared in-house by diluting the available Cefuroxime 750mg powder into balanced saline solution to reach the desirable concentration. Patients in the control group received only the 2 drops of topical moxifloxacin. Prophylactic antibiotics were not included in the irrigating solutions in either group. Over the 30 days following surgery, all patients were prescribed topical moxifloxacin eye drops for immediate postoperative use and a regimen of moxifloxacin 5.45 mg/ml eye drops associated with dexamethasone 1.00 mg/ml, regardless of their study group.

All patients underwent evaluations on the first postoperative day, the 15th day, and the 30th day. They were discharged from the surgery department after the 30-day follow-up and resumed their routine follow-ups at the service. The hospital maintains a 24-h ophthalmologic emergency room, and patients were instructed to seek care if necessary. During all follow-up appointments, a comprehensive ophthalmologic assessment was conduct, with retinal mapping performed only after 30 days. Any signs or symptoms suggestive of endophthalmitis, such as decreased visual acuity, conjunctival hyperemia, ciliary injection, eye pain, hypopyon, exacerbated inflammatory reaction, eyelid edema, surgical wound leakage, or positive Seidel's sign, were documented at each follow-up visit.

Statistical analyses were performed using Stata/SE Statistical Software, Release 14.0, 2015 (Stata Corp, College Station, Texas, USA). Frequency tables were used for descriptive analysis. Endophthalmitis rates were compared between the groups using Fisher's Exact test. Firth logistic regression was used to evaluate the effect of group on the endophthalmitis occurrence. *P* values ≤ 0.05 were considered statistically significant.

## Results

A total of 23,184 cataract surgeries were included in the current analysis. Control Group included 6207 procedures performed from January 2011 to December 2013. ATB-P Group included 16,977 procedures performed from January 2014 to December 2019.

During the entire study period, there were 9 reported cases of endophthalmitis (0.0388%), with 7 of those cases confirmed by positive culture (0.0302%). Table 1 shows the endophthalmitis occurrence according to the group.

**Table 1:** Endophthalmitis occurrence according to the group.

	Total surgeries	Reported cases	Positive culture	Estimated prevalence (%)	Prevalence confirmed by culture (%)
Control group (2011–2013)	6,207	6	6	0.0967	0.0967
ATB-P group 2014–2019	16,977	3	1	0.0177	0.0059

A significantly higher rate of endophthalmitis was observed in the control group when compared to the ATB-P group, for both reported (*p* = 0.014) and confirmed by culture (*p* = 0.002) cases. Surgeries performed with ATB prophylaxis showed 80% less chance of reported endophthalmitis (OR = 0.20; 95% CI 0.05–0.72; *p* = 0.014) and 92% less chance of confirmed by culture endophthalmitis (OR = 0.08; 95% CI 0.01–0.49; *p* = 0.006) than surgeries performed without the ATB prophylaxis.

In the control group, all the 6 cases confirmed by culture were positive for *Pseudomonas aeroginosa*, while the only case confirmed by culture in the ATB-P group was positive for *Staphylococcus epidermidis*. The *Staphylococcus epidermidis* strain found in the culture showed the following antibiogram profile: resistant to Cephalothin, Cefoxitin, Oxacillin, Ceftriaxone, Azithromycin, Penicillin; and sensitivity to Amikacin, Trimethoprim + Sulfamethoxazole, Linezolid, Gentamicin, Ciprofloxacin, Ofloxacin, Tobramycin, Vancomycin; and Intermediate behavior to Moxifloxacin.

Table 2 provides details on the diagnosis, treatment, and outcomes of endophthalmitis cases identified during the study period.

**Table 2:** Characteristics of diagnosis, treatment, and outcomes of the endophthalmitis cases.

	Group	Time until diagnosis (days)	Visual acuity at diagnosis	Treatment	Visual acuity post-treatment	Culture
Case 1	Control	2	LP	PPV + IV ATB	NLP	Positive
Case 2	Control	8	CF	PPV + IV ATB	20/60	Positive
Case 3	Control	2	LP	PPV + IV ATB	20/40	Positive
Case 4	Control	2	LP	PPV + IV ATB	20/20	Positive
Case 5	Control	2	HM	PPV + IV ATB	20/100	Positive
Case 6	Control	3	CF	PPV + IV ATB	NLP	Positive
Case 7	ATB-P	4	HM	PPV + IV ATB	20/30	Positive
Case 8	ATB-P	4	HM	PPV + IV ATB	20/30	Negative
Case 9	ATB-P	4	LP	PPV + IV ATB	20/30	Negative

*LP* light perception, *CF* counting fingers, *HM* hand movement, *NLP* no light perception, *PPV* Pars Plana Vitrectomy, *IV ATB* intravitreal antibiotics.

All the cases were diagnosed within 8 days of surgery, with very poor visual acuity. All cases from the ATB-P group achieved a visual acuity of 20/30, while cases in the control group had outcomes ranging from 20/20 visual acuity to no light perception.

## Discussion

Despite being a rare condition and of difficult causal determination, endophthalmitis is associated with some risk factors such as prolonged surgical time, intraoperative complications, diabetes status, surgeons with limited practical experience, service sterility breaches, among others^4,5,7,11^. Our institution has launched a cataract fellowship service in 2018, however, there has been no increase in the overall prevalence of infectious endophthalmitis since then. All cases presented in the current study were reported by experienced surgeons.

Our results demonstrate a reduction in the endophthalmitis incidence following the intraoperative use of intracameral cefuroxime, corroborating other studies on the same subject, with variable levels of association between the intervention and outcome^4,11,13,15,18–20^. The only prospective and randomized study on this subject was the ESCR study and, despite some bias associated with the non complete blinding designed, it is considered the most important one on the topic and it supports our findings^11^.

Our cataract surgeries consistently followed a protocol involving povidone-iodine eye drops, postoperative topical moxifloxacin, and moxifloxacin/dexamethasone eye drops for 30 days. Due to an increased number of infectious endophthalmitis cases in 2013, we introduced the use of antibiotic prophylaxis (ATB-P) with intracameral (IC) cefuroxime. While the literature supports the use of this drug, it is worth noting that it has some limitations including potential associations with complications derived from contamination, dilution errors, toxic anterior segment syndrome, and macular toxicity^18^. Furthermore, the drug has limited action on Gram negatives, including some strains of Serratia, some strains of Proteus and *Bacteroides fragilis*, and inactivity on Pseudomonas^15^. There are still references in the literature reporting worse visual results associated with the cefuroxime ATB-IC use, largely due to increased rates of infections with gram-negative cefuroxime-resistant strains^11^. In our work, we did not find gram-negative infection after the introduction of ATB-P but we noticed a delay in the diagnosis of endophthalmitis by one day in the ATB-IC group when compared to the control cases.

We did not consider the intracameral use of moxifloxacin or vancomycin due to potential toxicity and local regulations. The available formulations of moxifloxacin in Brazil contain preservatives and adjuvants that are toxic to ocular structures^20^; even the most used eye drops in our country (Vigamox®, Alcon), market as not having preservatives, has a warning that the solution should not be injected under the conjunctiva, nor introduced directly into the anterior chamber of the eye^20,21^. Other studies performed in other locations have, however, shown good results on its use, safety, and efficacy^16,22^. Vancomycin has no action on gram-negatives and has been associated with occlusive retinal vasculitis and, although few cases, its use was considered imprudent. In addition, the FDA recommends not to use vancomycin for prevention of endophthalmitis, and limits its use for the treatment of infection itself, especially in patients at risk for Methicillin-resistant *Staphylococcus aureus*, Methicillin-resistant *Staphylococcus epidermidis*^12,21,23^. A recent metanalysis comparing different intracameral injection has shown vancomycin (OR = 0.03, 99.6% CI 0.00–0.53, *p* value = 0.006) as the best preventive effect on preventing endophthalmitis, followed by intracameral injection of cefuroxime (OR = 0.18, 99.6% CI 0.09–0.35, *p* value < 0.001), and moxifloxacin (OR = 0.36, 99.6% CI 0.16–0.79, *p* value = 0.003)^16^. In any case, the local health regulations in Brazil do not advice the use of intracameral vancomycin or moxifloxacin.

One of our cases (case #7) was confirmed as infectious endophthalmitis by gram-positive and showed an antibiogram profile resistant to most cephalosporins. This case involved a male, elderly patient, insulin-dependent diabetic, and immunosuppressed due to metastatic neoplasia (breast and lung cancer). The bacterial resistance is related to an intrinsic property of a bacterial species or an acquired capacity, induced by mutation in native DNA or introduction of resistant DNA that can be transferred between different genera or species^24^. Moreover, in immunosuppressed patients there is population/colonization by species other than the usual ones, especially when they undergo prolonged hospitalization, which may explain the observed resistance.

The discussion on antibiotic use and bacterial resistance is ongoing. Cefuroxime resistance mechanisms include beta-lactamase hydrolysis, reduced penicillin-binding protein affinity, outer membrane impermeability in Gram-negative bacteria, and bacterial drug efflux pumps^25^. It is also known that cefuroxime exhibits time-dependent antibacterial activity, so that it needs to exceed a minimum duration (about 2 to 3 h) of exposure to the drug, in concentrations 4 to 6 times the minimum inhibitory concentration to obtain greater antibiotic efficacy and a lower risk of developing resistance^26^. There is also experimental evidence that suggest that exposure to antibiotics for less than 2 h may not be adequate to inhibit the growth of organisms that commonly cause postoperative infectious endophthalmitis^26^.

According to the concept of antibiotic prophylaxis, the maximum time of exposure to antibiotic should be up to 24 hours^3,5–7^, as a longer period would not bring benefit to the patient and would promote the risk of inducing bacterial resistance^2^, in addition to increase the risk of adverse events^1^ and costs^15,27^. After the intracameral injection of 1mg of cefuroxime, the levels of antibiotics in the aqueous humor remain above the inhibitory concentration for several relevant bacterial species up to 4–5 h after surgery^25^. Under usual conditions, the aqueous humor formation rate is 2.5–2.8 µL/min and this volume is completely replaced every 100 min^28^. Moreover, the turnover of cefuroxime injected into the chamber is approximately 8 h, for a concentration of 40 µg/20 µL (0.04 mg/0.02 ml)^29^. Taking in consideration that cefuroxime turnover follows a linear pattern of clearance regardless of injection sites (systemic versus ocular)^29^ and even though aqueous humor (AH) homeostasis is modified by ocular inflammation, possibly the total volume injected into the eye of cefuroxime 1 mg / 0.1 ml, is not completely eliminated within 24 hours. However, due to the low concentration injected, due to the tiny residual concentration stored in the anterior chamber and in the capsule, due to the despicable levels reached systemically, and the ethical consideration on risks related to increased risk of inducing resistance microbial activity against the benefits would justify its use.

Patients from the current study with suspected infectious endophthalmitis in the postoperative period were directed to diagnostic confirmation. All subjects underwent through ocular ultrasound and vitrectomy via pars plana with intravitreal injection of antibiotics of Ceftazidime (2.25 mg/0.1 ml) and vancomycin (1.0 mg/0.1 ml), even those with visual acuity better than light perception, as recommended by the EVS^1^. In all cases, vitreous biopsies were collected and sent for staining and culture. The diagnosis of endophthalmitis is clinical and the treatment should not be delayed when signs and symptoms are indicative, as it may be occurring rapid destruction of ocular tissues and only after the third day of intraocular infection that pathogen-specific antibodies can be detected^12^. Consequently, laboratory results of staining and culture can be negative while severe inflammation occurs in the eye.

The prognosis for cases with negative culture is likely to be good, as sterile inflammation in the absence of bacterial toxins minimizes the risk of retinal damage^30^. In cases of positive culture, the severity of the intraocular infection depends on factors like inoculum, microorganism virulence, host immune responses, perioperative measures, and timing of infection presentation^12^. This justifies the different visual results found in the cases from 2013, as patients with early diagnosis and treatment for the virulent microorganism evolved to good visual acuity (cases #3 and #4) as opposed to patients who evolved to total blindness (cases #1 and #6) likely due to the different amount of inoculum; while one patient with a relatively late diagnosis and treatment (i.e. eighth postoperative day), evolved with low vision (case #2).

Furthermore, even with early diagnosis and institution of adequate treatment, the visual prognosis tends to be poor, as toxins produced by the microorganism and host inflammatory responses cause rapid damage to the photoreceptor and these effects can remain even after the contents eyepieces have become sterile^30^. Approximately fifty percent of affected patients do not restore vision of 20/40 or better and about thirty percent have visual acuity worse than 20/200 after treatment^1,7,30^. The cases developed prior the use of ATB-P of cefuroxime IC follow similar outcomes in terms of vision, but all the cases occurring after the introduction of ATB-P have restore vision to 20/30.

Despite the substantial sample size in the current study, there are some limitations worth noting. Firstly, there were no cases of gram-negative bacterial infections in our dataset, which may potentially overestimate the beneficial effects of intracameral antibiotic prophylaxis with cefuroxime. Additionally, in our pursuit of maximizing the sample size, we collected data from surgeries performed by a large number of surgeons. This also contributes to other potential limitations associated with the retrospective study design, including the risk of selection bias, concerns about data quality, and the presence of confounding variables. For future studies investigating the use of intracameral antibiotic prophylaxis in cataract surgeries, randomized controlled trials are advisable to address these limitations more effectively.

In conclusion, our findings indicate that the use of povidone-iodine eye drops associated with ocular topical antibiotics in the immediate postoperative period was not enough to avoid the occurrence of acute infectious endophthalmitis. After the addition of intracameral antibiotic-prophylaxis with cefuroxime into the regular protocol, the rates of infectious have drastically dropped into acceptable values within a high-volume surgical service. Considering that the conjunctiva is not sterile, the use of an ocular prosthesis (intraocular lens), the poor visual prognosis related to a postoperative acute infectious endophthalmitis, the high costs associated with treating an infectious endophthalmitis in comparison to the low cost of prophylaxis, and the visual and psychological morbidity, we strongly recommend the use of antibiotic prophylaxis to prevent infectious endophthalmitis in the postoperative period of cataract surgery.

## Data Availability

The data that support the findings of this study are available from Ophthal Hospital Especializado but restrictions apply to the availability of these data, which were used under license for the current study, and so are not publicly available. Data are however available from the corresponding author upon reasonable request and with permission of Ophthal Hospital Especializado.

## References

[1] Endophthalmitis Vitrectomy Study Group (1995). A randomized trial of immediate vitrectomy and of intravenous antibiotics for the treatment of postoperative bacterial endophthalmitis. Arch. Ophthalmol..

[2] Oréfice F, Freitas Neto CA, Alves MR (2013). Uveítes 3 ed. CBO.

[3] Kresloff MS, Castellarin AA, Zarbin MA (1998). Endophthalmitis. Surv Ophthalmol..

[4] Cao H, Zhang L, Li L, Lo S (2013). Risk factors for acute endophthalmitis following cataract surgery: A systematic review and meta-analysis. PLoS One..

[5] Keay L, Gower EW, Cassard SD, Tielsch JM, Schein OD (2012). Postcataract surgery endophthalmitis in the United States: Analysis of the complete 2003 to 2004 Medicare database of cataract surgeries. Ophthalmology.

[6] Levin ASS (2002). Quais os princípios gerais da profilaxia antibióti-ca antes de intervenção cirúrgica?. Rev. Assoc. Med. Bras..

[7] Vaziri K, Schwartz SG, Kishor K, Flynn HW (2015). Endophthalmitis: State of the art. Clin. Ophthalmol..

[8] Kernt M, Kampik A (2010). Endophthalmitis: Pathogenesis, clinical presentation, management, and perspectives. Clin. Ophthalmol..

[9] Speaker MG, Menikoff JA (1991). Prophylaxis of endophthalmitis with topical povidone-iodine. Ophthalmology.

[10] Ciulla TA, Starr MB, Masket S (2002). Bacterial endophthalmitis prophylaxis for cataract surgery: An evidence-based update. Ophthalmology.

[11] Haripriya A (2017). Antibiotic prophylaxis in cataract surgery—an evidence-based approach. Indian J. Ophthalmol..

[12] Barry P, Cordovés L, Gardner S. *ESCRS Guidelines for Prevention and Treatment of Endophthalmitis Following Cataract Surgery: Data, Dilemmas and Conclusions. The European Society for Cataract & Refractive Surgeons.* https://www.escrs.org/media/uljgvpn1/english_2018_updated.pdf (2013).

[13] Endophthalmitis Study Group (2007). Prophylaxis of postoperative endophthalmitis following cataract surgery: Results of the ESCRS multicenter study and identification of risk factors. J. Cataract Refract. Surg..

[14] Neu HC, Fu KP (1978). Cefuroxime, a beta-lactamase-resistant cephalosporin with a broad spectrum of gram-positive and -negative activity. Antimicrob. Agents Chemother..

[15] Barry P (2006). ESCRS study of prophylaxis of postoperative endophthalmitis after cataract surgery: Preliminary report of principal results from a European multicenter study. J. Cataract Refract. Surg..

[16] Kato A (2022). Prophylactic antibiotics for postcataract surgery endophthalmitis: A systematic review and network meta-analysis of 6.8 million eyes. Sci. Rep..

[17] Durand ML (2013). Endophthalmitis. Clin. Microbiol. Infect..

[18] Bowen RC (2018). Comparative analysis of the safety and efficacy of intracameral cefuroxime, moxifloxacin and vancomycin at the end of cataract surgery: A meta-analysis. Br. J. Ophthalmol..

[19] Kessel L, Flesner P, Andresen J, Erngaard D, Tendal B, Hjortdal J (2015). Antibiotic prevention of postcataract endophthalmitis: A systematic review and meta-analysis. Acta Ophthalmol..

[20] Alves MR, Victor G, Carricondo PC, Nose RM (2017). Moxifloxacino, cefuroxima e vancomicina intracameral: Espectro de atividade, preparo, segurança e eficácia. Rev. Dig. Oftalmol..

[21] Lucena NP, Pereira IMS, Gaete MIL, Ferreira KSA, Mélega MV, Lira RPC (2018). Intracameral moxifloxacin after cataract surgery: A prospective study. Arq. Bras. Oftalmol..

[22] Haripriya A, Chang DF, Ravindran RD (2019). Endophthalmitis reduction with intracameral moxifloxacin in eyes with and without surgical complications: Results from 2 million consecutive cataract surgeries. J. Cataract Refract. Surg..

[23] Food and Drug Administration FDA. *Vancomycin Injection*. https://www.accessdata.fda.gov/drugsatfda_docs/label/2017/050671s024lbl.pdf (2017).

[24] Reygaert WC (2018). An overview of the antimicrobial resistance mechanisms of bacteria. AIMS Microbiol..

[25] Laboratoires Théa, Clermont-Ferrand, France. *Aprokam*® https://www.medicines.org.uk/emc/medicine/27397#gref (2020).

[26] Arshinoff SA, Felfeli T, Modabber M (2019). Aqueous level abatement profiles of intracameral antibiotics: A comparative mathematical model of moxifloxacin, cefuroxime, and vancomycin with determination of relative efficacies. J. Cataract Refract. Surg..

[27] Pires MR (2012). Avaliação do uso de cefazolina como profilaxia antibiótica em procedimentos cirúrgicos. Rev. HCPA.

[28] Vaajanen A, Vapaatalo H (2011). Local ocular renin-angiotensin system - a target for glaucoma therapy?. Basic Clin. Pharmacol. Toxicol..

[29] Jairam RK (2020). Uptake and pharmacokinetics of cefuroxime in rabbits after intravitreal, intracameral, and topical dosing: Relevance to human ocular injection of cefuroxime. Xenobiotica.

[30] de Geus SJR, Hopman J, Brüggemann RJ, Klevering BJ, Crama N (2021). Acute endophthalmitis after cataract surgery: Clinical characteristics and the role of intracameral antibiotic prophylaxis. Ophthalmol. Retina.

